# Absent Treatment

**Published:** 1905-10

**Authors:** 


Absent Treatment.—A voice from the grave (or elsewhere; present address not given), Mrs. Pinkham, Lydia E. Pinkham, inventor of the "justly celebrated Vegetable Compound," whose picture and signature "Yours for Health," are so familiar to newspaper readers, is *dead.* She has been dead twenty-two years. But she is still treating female complaints with her Vegetable Compound. The following advertisement bears date of June 27, 1905:

"Years ago Mrs. Pinkham, at Lynn, Mass., determined to step in and help her sex. Having had considerable experience in treating female ills with her Vegetable Compound, she encouraged the women of America to write to her for advice in regard to their complaints, and being a woman, it was easy for her ailing sisters to pour into her ears every detail of their suffering. * * * * *

"No physician in the world has had such a training, or has such an amount of information at hand to assist in the treatment of all kinds of female ills. This, therefore, is the reason why Mrs. Pinkham, in her laboratory at Lynn, Mass., is able to do more for the ailing women of America than the family physician. Any woman, therefore, is responsible for her own suffering who will not take the trouble to write to Mrs. Pinkham for advice."

If this is not fraud, it would be hard to define fraud. It is up to the postoffice department to look after this advertisement.

---

A Shame and an Outrage if not a crime was the printing by the New Orleans *Times-Democrat* of that alleged "Interview with Dr. Hartman" wherein the people were told that the way to prevent and cure yellow fever was to take and keep on taking Peruna. It sold millions of bottles of the stuff, no doubt. Was there ever such infamy? playing upon the fears of a stricken people to extort money from them. It is an alcoholic drink that in many persons it is said to have fixed the drink habit. It is sold in prohibition towns for "what's in it." The United States Commissioner of Revenue has tabooed it as will be seen by the order elsewhere printed—recently promulgated. Here is what *Collier's Weekly* says of it. Collier deserves the thanks of all decent people, everywhere:

MURDERED BY ADVERTISEMENT.

Patent medicine horrors never reached a point of deeper degradation than in the yellow fever troubles of the South. Mr. Samuel H. Adams, whose series of articles will begin probably in five or six weeks, will hardly have anything more startling to narrate than the incredible performance of "Peruna" in alliance with the New Orleans *Times-Democrat*. This sheet has accomplished a feat of prostitution which, considering its pretense to respectability, probably sets the record. While the South is struggling to check a peril of the direst magnitude, this newspaper publishes an interview with "Dr. Hartman," with the familiar allegation that he "said in part," and all other devices to make it look like an important piece of news. Its headlines are: "How to Avoid Yellow Peril. An Interview with Dr. Hartman Concerning the Yellow Plague." To the reader this is the genuine opinion of a physician. He can not know that Dr. Hartman is the head of the Peruna Company, and that the *Times-Democrat*, in whom the reader presumably has some trust, is selling itself and the safety of its constituents for a bag of gold. "A summary of this interview," the *Times-Democrat* informs us, "is being spread broadcast over the United States *for the benefit of yellow fever sufferers.*" The gist of it is that, while screens and other precautions are advisable, Peruna should be taken at once and continued during the whole course of the epidemic. "'I feel sure,' the doctor went on to say (!), 'that any person following this advice is in no danger of taking yellow fever.'" For anybody who believes we have taken too seriously the patent medicine evil and newspaper complicity therein, this unspeakable outrage should be a lesson. Is there anything to which men can not by led by money? To own a newspaper and hire it out to perilous fraud in an emergency like the yellow fever danger almost surpasses one's belief in human greed. No more disheartening proof of the need of the crusade which we have begun could possibly have been offered.

